# Percutaneous closure of perimembranous and infundibular ventricular septal defects: single-center experience in 203 patients with medium- and long-term follow-up

**DOI:** 10.1016/j.ijcchd.2025.100641

**Published:** 2025-11-11

**Authors:** Nataliia Yashchuk, Igor Ditkivskyy, Denys Voloshyn, Bogdan Cherpak, Yuliia Yermolovych

**Affiliations:** a Department of Interventional Cardiology for Congenital and Acquired Heart Disease, Amosov National Institute of Cardiovascular Surgery, 6 Amosov Street, 03038, Kyiv, Ukraine; b Department of Pediatric Cardiac Surgery, Amosov National Institute of Cardiovascular Surgery, Kyiv, Ukraine

**Keywords:** Ventricular septal defect, AV block, Aortic insufficiency, Tricuspid regurgitation, Percutaneous closure, Residual shunt

## 1 Background

Ventricular septal defect (VSD) is the most common congenital heart defect. According to the location of the defect in the ventricular septum, it can be categorised into the muscular (mVSD), inlet, perimembranous (pmVSD)with either inlet or outlet extension, outlet and infracristal (infundibular) types. The most commonly diagnosed type in general population, accounting for approximately 80 % of all VSDs, is perimembranous VSD [1]. It is located in the membranous part of the septum with the possible extension into the inlet or outlet septum; adjacent to the aortic or tricuspid valves; often is associated with so-called aneurysms of the septal leaflet of the tricuspid valve (TV) [1]. Transcatheter device closure of pmVSD has been widely adopted in clinical practice [[2], [3], [4]]. There has been growing interest regarding the long-term efficacy and safety. Furthermore, the feasibility of transcatheter closure of infracristal or subpulmonary (VSD) and pmVSD with outlet extension – including those with deficient aortic rim or mild pre-existing aortic regurgitation (AR) and utilisation of zero asymmetric devices, has been reported in a few large centres in China [[5], [6], [7]]. However, the data regarding the follow-up of percutaneous closure of these defects are still limited. This study was designed to assess the medium and long-term follow-up of transcatheter closure of perimembranous with inlet or outlet extensionand infracristal VSDs in a high-volume center.

## 2 Materials and methods

**Fig. 1:**
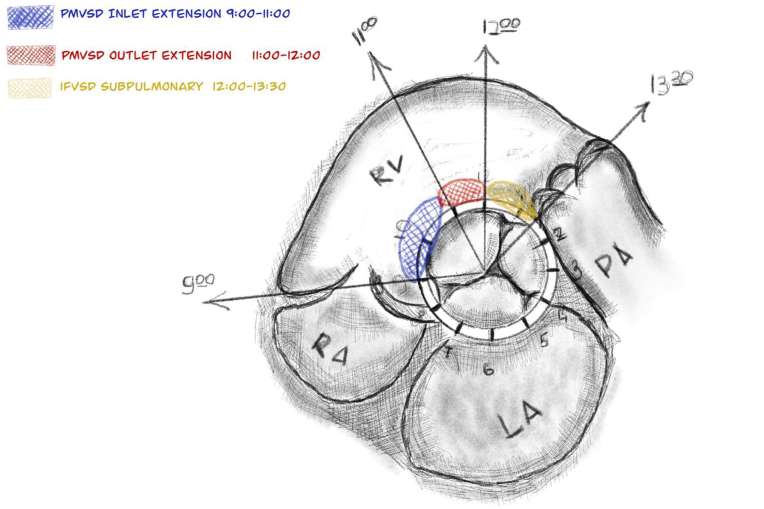
Diagrammatic representation of possible VSD locations on standard echo short axis view at aortic valve level.

A total 203 consecutive patients who underwent transcatheter VSD closure between August 2012 and January 2025 were retrospectively analysed. Among them, 166 pts (81,78 %) had perimembranous VSD (pmVSD)central or with inlet extension; 31 pts (15,27 %) – pmVSD with outlet extension and 6 pts (2,95 %) had infracristal or subpulmonary VSD (ifVSD). Patients who underwent hybrid VSD closure were excluded from the study. Prior to the procedure, all patients underwent transthoracic echocardiography (TTE) and were evaluated by the standard echocardiographic protocol. Location, morphology, and the size of the defect, presence of ventricular septal aneurysm, end-diastolic diameter Z-score of the left ventricle (LVEDD Z-score), left atrium to ascending aorta ratio (LA/Ao), and pre-existing aortic (AR) and/or tricuspid regurgitation (TR) were recorded. A diagrammatic representation of possible VSD locations on standard echo views is shown in Fig. 1 [5,8,9]. Defects located at 9 to 11 o'clock in the short axis parasternal TTE view were defined as pmVSD with inlet extension, defects located at 11 to 12 o'clock were defined as pmVSD with outlet extension and from 12 to 1:30 were defined as icVSDs (subpulmonary).

Additional defect assessment on transoesophageal ECHO (TEE) was provided in all patients since 2017. The LAO 30°CRA 20° angiographic projection was preferred in all patients. The distance to the aortic valve was assessed in five-chamber TTE view, 110–130° TEE view, and angiographically. The aortic rim less than 2 mm in a case without ventricular aneurysm was considered to be deficient, but it was not an absolute contraindication for percutaneous VSD closure. A soft, thin, low-profile nitinol mesh occluder such as MFO or ADO II would adapt itself to the moving leaflets of the aortic valve with minimal interference of the leaflet mobility, at least in the short term [10,11]. Our clinical observations indicated that cases suspected of a deficient rim to the aortic valve on TTE or TEE data were frequently disapproved on angiographic assessment. We believe that aortic rim is a three-dimensional structure that may not be adequately visualized on 2D echocardiography. However, its presence could potentially be confirmed by 3D ECHO (not available in our institution) or by angiography. All defects with the rim more than 5 mm were classified as a high muscular VSD. The **inclusion criteria** were as follows: clinical symptoms such as growth retardation or recurrent respiratory infection in children; hemodynamically significant VSD (left ventricle overload (LVEDD z-score ≥2 or LVEDVI≥75 ml/m2) and/or left atrium enlargement LA/Ao >1,5), history of infective endocarditis [2,3,5]. **Exclusion criteria included**: 1. Dextraposition of the aorta 2. Subaortic or subpulmonary stenosis 3. Aortic valve prolapse with progressive aortic regurgitation (more than mild) in patients older than 18 years old 4. Inlet VSD 5. Eisenmenger syndrome 6. Aortic rim less than 2 mm in patients older than 18 years old 7. Defect size more than 16 mm 8. Infective endocarditis [[2], [3], [4], [5], [6], [7]].

The Ethics Committee of Amosov National Institute of Cardiovascular Surgery approved the study protocol. The Study complied with the declaration of Helsinki.

## 3 Devices

In this study, severaltypes of devices were employed for transcatheter VSD closure. These included - Amplatzer-like muscular Ventricular Septal Occluders (MVSO) devices: (MemoPart mVSD – Shanghai Shape Memory Alloy, Shanghai, China; Lepu mVSD LifeTech Scientific, Shenzhen, China); Hyperion MVSO (COMED B.V, Holland); original MVSO – Abbott vascular, USA); Nit-occlud Le VSD and Nit-occlud PDA (PFM medical, Cologne, Germany); KONAR-MF – multifunctional (LifeTech Scientific, Shenzhen, China); Amplatzer-like PDA occluder Aduct Duct Occuder type I (ADOI) (MemoPart PDA – Shanghai Shape Memory Alloy, Shanghai, China; Cera PDA LifeTech Scientific, Shenzhen, China; original Amplatzer Duct occluder I – Abbott vascular, USA). Thin-waist and asymmetric devices were deliberately avoided in our series due to their reported association with a higher incidence of atrioventricular block (A-V block) [5,[12], [13], [14], [15]]. We do not use ADO II devices, be because they are unavailable in our country.

Device selection was guided by anatomical characteristics and the presence of ventricular septal aneurysm. Before 2016 we have utilized only Nit-occlude le VSD devices in all paediatric and some adult patients with pmVSD accompanied with septal aneurysm. Coil diameters 1–2 mm larger than the maximum aneurysm diameter were selected. In adult patients with tubular-shaped and window-type VSDs, or when the entry point to the aneurysm was narrower than the aneurysm itself, we used MVSO devices with a size 1–2 mm larger than the entry side (Fig. 3a and b; Fig. 5A and B). Since the introduction of the KONAR-MF device in 2016, the Nit-Occlude Le VSD has been used less frequently. It presents several significant disadvantages compared to the KONAR-MF device: a larger delivery sheath profile, only antegrade implantation is possible, and the procedure is more time-consuming. In the KONAR-MF era, we exclusively used the KONAR-MF in all pediatric patients, and AVSO devices for tubular-shaped and window-type VSDs or when the aneurysm entry point was smaller than the maximum aneurysm diameter in adults. KONAR-MF device has one significant disadvantage – right side disk is staying apart from the left part of the device and misaligned with the ventricular septum. It interfered with the closure mechanism of the tricuspid valve, leading sometimes to severe tricuspid regurgitation. Therefore, in cases involving a septal aneurysm, the degree of tricuspid regurgitation guided the device's implantation site. Repositioning was sometimes necessary, with several approaches considered based on the aneurysm's specific morphology: 1. Device stent the entry to the aneurysm (Fig. 3b); 2. Device stent the defect in aneurysm (Fig. 3c); 3. Device filled the aneurysm (Fig. 3d, e, f) – this approach was preferred especially in cases of septal aneurysm with multiple exits. When it was necessary to fill the aneurysm (especially in aneurysms with multiple exits), the size of the left disk was equal to or 1 mm larger than the maximum aneurysm diameter.

### 3.1 Procedure

The procedure was performed under general or local anesthesia (depending on the age and TEE probe utilisation). Since 2017, all procedures have been conducted under continuous TEE guidance to monitor for the new-onset of TR or aortic insufficiency (AI). Two standard approaches were utilized: the transvenous (antegrade) and transarterial (retrograde), as previously described in detail in earlier publications [3,12,[14], [15], [16], [17], [18]]. Retrograde approach was preferred since 2018.

TEE was systematically applied prior to the device release to ensure absence no new-onset TR and/or AI.

**Fig. 2:**
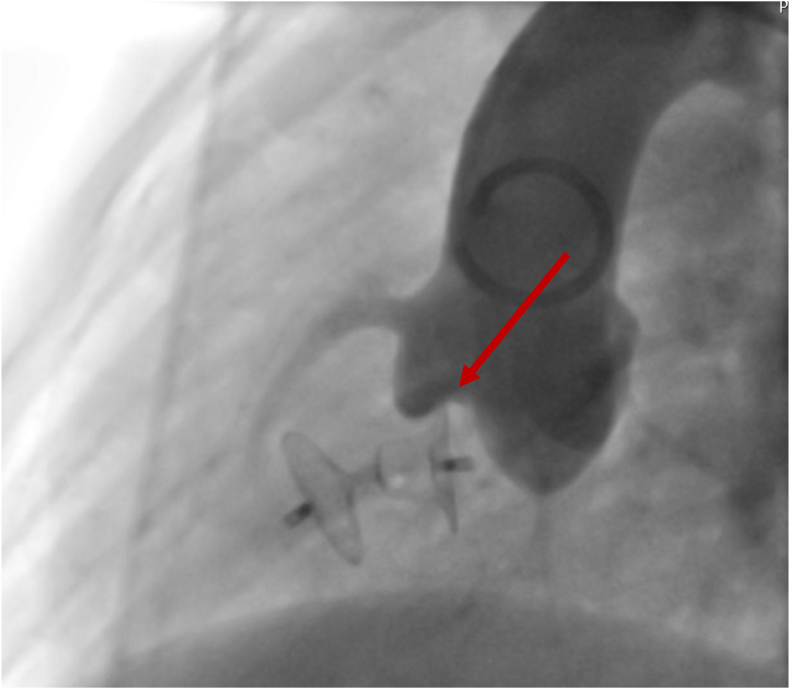
Angiography from the ascending aorta. Aortic valve pinching with KONAR-MF occluder.

During the procedure the presence of aortic valve “pinching” (Fig. 2), new-onset TR or AI, residual shunt and other complications were meticulously recorded when detected.

**Fig. 3:**
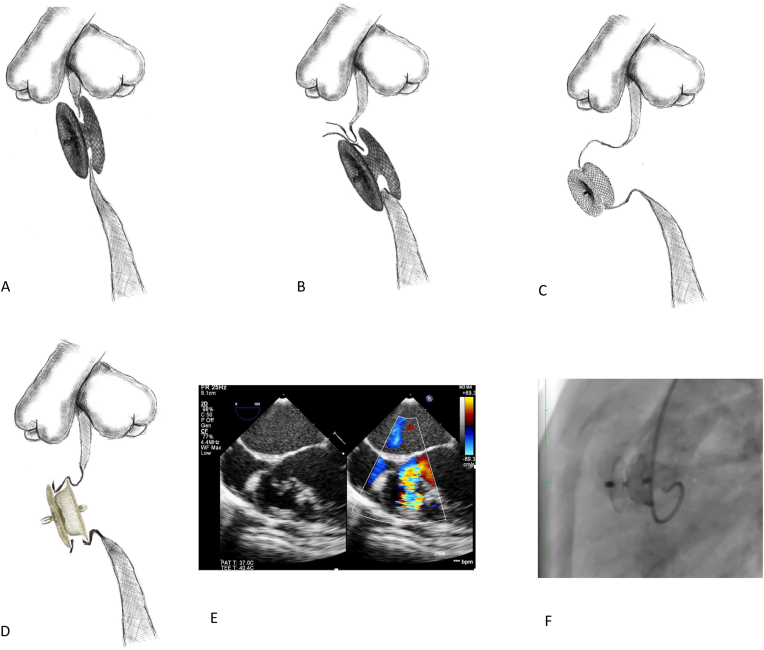
Schematic picture of device position. **A,b,c** -device stent the defect or the entry to the aneurysm **d**-device filled the aneurysm **e,f** - 4 month old boy 5,6 kg device filled the aneurysm.

All patients underwent ECG and TTE 1 day after the procedure. In cases of rhythm disturbances Holter ECG was performed. In absence of adverse events, patients were discharged the following day. Aspirin was prescribed at a dosage of 3–5 mg/kg daily for three months to all patients.

Residual shunt was categorised into mild (1 mm–2 mm), moderate (2 mm–4 mm), or severe (>4 mm). The presence of flow through the device was called intradevice shunt and was considered less significant compared to mild shunt [19].

All complications were categorised into major and minor complications.

*Major complications*: death, fatal adverse events, events requiring surgery (embolization, myocardial perforation, TV chordae rupture, severe residual shunt, severe haemolysis, valvular damage, persistent AVB).

*Minor complications*: complications that resolve spontaneously or with medical therapy and don't have fatal outcomes (vascular complications, mild haemolysis solved with medical therapy, complete transient AVB or other conduction disturbances that do not require pacemaker implantation or surgical device retrieval, device embolization or dislodgement with transcatheter device retrieval) [5,12,19].

The procedural success was defined by absence of major complications or severe residual shunt with the stable device position in the VSD [5,12,19].

## 4 Follow-up protocol

All patients underwent scheduled follow-up evaluation including physical examination, electrocardiography (ECG) and TTE scheduled at 1, 3, 6, 12, 24, and 36 months after the procedure and annually thereafter. In addition, 24-h Holter monitoring was performed for patients who presented with postprocedural atrioventricular block or LBBB at any outpatient examination. Follow-up data were collected up to January 2025.

## 5 Statistics

The data were expressed as numbers or percentages for categorical variables as mean ± SD. The difference between groups in case of quantitative numerical characteristics was calculated using the Mann-Whitney test, the difference in qualitative characteristics (% ratio) between groups was calculated using the Pearson χ2 test, and the difference between groups was considered significant at p < 0.05. P_1-2_ was described as a difference between groups 1 and 2; P_1-3_ – between groups 1 and 3 and P_2-3_ – between groups 2 and 3 correspondingly.

Correlation analysis was performed using Spearman, the correlation was defined as weak when the R coefficient was below 0.25, medium strength with the R coefficient of 0.26–0.5, and strong when it was above 0.51.

## 6 Results

The mean age of the patients was 13,1 ± 1,1 year (from 4 months to 63 years old). Of the patients, 18 were up to 1 year old, 96 patients were from 1 to 6 years old, 41 patients were from 6 to 18 years old and 48 patients were older than 18 years old. The mean weight of the patients was 33,58 ± 1,9 (median 19 kg; from 5,6–116 kg). The mean size of the defect, as assessed by angiography, was 4,84 ± 5,67 mm. In patients without aneurysmal tissue, the mean distance to the aortic valve was 2,4 ± 1,44 mm. In 138 pts, the device directly stented the defect in tubular-shape or window type VSDs (Fig. 3a), entry to the aneurysm (Fig. 3, Fig. 5 A, B) or rupture in the aneurysm (Fig. 3c); while in 61 pts, the device was deployed within the aneurysmal tissue and filled the aneurysm (Fig. 3d–f). Fifteen patients had residual ventricular septal defects (VSD) closed percutaneously following surgery. Nine of these patients had undergone VSD repair (patch or suture), two had undergone Tetralogy of Fallot repair, and two had Gerbode-type VSDs (one following atrioventricular canal repair and one after mitral valve replacement). One patient had an artificial VSD created after myectomy for hypertrophic cardiomyopathy, and one patient had a residual VSD following a hybrid VSD closure.

Detailed baseline characteristics, stratified by anatomical types (pmVSD inlet extension, pmVSD outlet extension and icVSD) are summarized in Table 1.

**Table 1:** Baseline characteristics of the patients.

	pmVSD with inlet extension	pmVSD with outlet extension	icVSD	р
	N = 166 (81,78 %)	N = 31 (15,27 %)	N = 6 (2,95 %)	
Congenital	155 (93,3)	28 (90,3 %)	5 (83,3 %)	Р1-2 = 0,37 р2-3 = 0,56 Р1-3 = 0,12
Residual (postsurgery)	11 (6,7 %)	3 (9,7 %)	1 (16,7 %)	
Ventricular septal aneurysm	107 (64,4 %)	7 (22,6 %)	0	Р1-2 = 0,001
				р2-3 = 0,32
				Р1-3 = 0,014
Multiple exits	39 (23,5 %)	4 (12,9 %)	1 (16,7 %)	Р1-2 = 0,19
				р2-3 = 0,34
				Р1-3 = 0,56
Age (years)	13,6 ± 1,0	9,6 ± 1,2	16,8 ± 2,2	Р1-2 = 0,21
				р2-3 = 0,19
				Р1-3 = 0,64
Weight (kg)	33,7 ± 1,4	29,3 ± 2,4	49,0 ± 5,3	Р1-2 = 0,44
				р2-3 = 0,1
				Р1-3 = 0,18
Number of patients under 6 years of age	93 (56,0 %)	19 (61,3 %)	2 (33,3 %)	Р1-2 = 0,69
				р2-3 = 0,18
				Р1-3 = 0,34
Size of the defect in mm	4,8 ± 0,2	3,8 ± 0,3	5,0 ± 0,9	Р1-2 = 0,39
				р2-3 = 0,09
				Р1-3 = 0,97
**Aortic valve abnormalities**				
-bicuspid AV	2 (1,2 %)	7 (22,5 %)	0	Р1-2 = 0,001
				р2-3 = 0,45
				Р1-3 = 0,46
-AI before procedure	3 (1,8 %)	3 (9,6 %)	0	Р1-2 = 0,016
				р2-3 = 0,54
				Р1-3 = 0,19
-AV prolapse	0	2 (6,5 %)	0	Р1-2 = 0,06
				р2-3 = 0,34
Mean PA pressure ≥20 mmHg	25 (15,1 %)	4 (12,9 %)	1 (16,7 %)	Р1-2 = 0,76
				р2-3 = 0,48
				Р1-3 = 0,78
Ao/LA ratio	1,42 ± 0,2	1,43 ± 03	1,3 ± 0,3	Р1-2 = 0,40
				р2-3 = 0,42
				Р1-3 = 0,39
Distance from AV in a patient without aneurysm, mm	2,9 ± 0,2	1,6 ± 0,2	1,8 ± 0,3	P1-2 = 0,00001
				P2-3 = 0,57
				P1-3 = 0,31

The overall procedural success was 97,04 % (197 from 203 pts). The incidence of major complications was 1,97 % (n = 4), while minor complications occurred in 17,24 % (n = 35). Procedural success did not significantly depend on the anatomical type of VSD. However, weak negative correlations were observed between procedural success and the following factors.

• body surface area (BSA) (R coefficient = - 0,14, p = 0,03),
• antegrade closure (R coefficient = −0,24, p = 0,0003), and
• coil device usage (R coefficient = - 0,13, p = 0,04).

Conversely, a weak positive corelation was found between procedural success and the use of KONAR-MF device (R coefficient = 0,24, p = 0,0007).

The general characteristics of the groups are listed in Table 2.

**Table 2:** Detailed results of pmVSD and icVSD percutaneous closure.

	PmVSD with inlet extension N = 166	PmVSD with outlet extension N = 31	icVSD N = 6	P value
Procedure successful	162 (97,6 %)	29 (93,5 %)	6 (100 %)	Р1-2 = 0,22
				р2-3 = 0,87
				Р1-3 = 0,89
Procedure not successful	4 (2,4 %) (1-chordae rupture, 1-migration to PA, 2-device dislocation-surgery)	2 (6,5 %) (1-device dislocation,1- severe AI)	0	Р1-2 = 0,22
				р2-3 = 0,87
				Р1-3 = 0,88
-antegrade	57 (34,3 %)	7 (22,5 %)	3 (50 %)	Р1-2 = 0,19 р2-3 = 0,08 р2-3 = 0,08
-retrograde	109 (65,6 %)	24 (77,4 %)	3 (50 %)	
**Devices:**				
MVSO	24 (14,5 %)	4 (12,9 %)	2 (33,3 %)	
Coils	37 (22,2 %)	2 (6,5 %)	0	
KONAR-MF	104 (62,6 %)	24 (77,4 %)	3 (33,3 %)	
PDA occluders	1 (0,7 %)	1 (3,2 %)	1 (16,5 %)	
Occluder “pinching” AV	14 (8,4 %)	24 (77,4 %)	1 (16,6 %)	Р1-2 = 0,0001
				р2-3 = 0,11
				Р1-3 = 0,48
**Mayor complications** **Total:**	1 (0,6 %)	1 (3,22 %)	0	Р1-2 = 0,18
				р2-3 = 0,77
				Р1-3 = 0,89
Severe TR			0	
Severe AI	1 (0,7 %)	1 (3,22 %)	0	
**Minor complications** **Total:**	28 (16,87 %)	5 (16,12 %)	3 (50 %)	Р1-2 = 0,07
				р2-3 = 0,015
				Р1-3 = 0,03
**TR after procedure:**				
-mild	10 (6,0 %)	2 (6,5 %)	0	
-moderate	14 (8,4 %)	1 (3,2 %)	0	
**TR 1 month after procedure**				P1-2 = 0,57 P2-3 = 0,55
-mild	12 (7,2 %)	1 (3,2 %)	0	P1-3 = 0,44
-moderate	3 (1,8 %)	0	0	P1-2 = 0,76 P1-3 = 0,57
**AI**				
**-**mild	2 (1,2 %)	2 (6,5 %)	1 (3,2 %)	
-moderate		1 (3,22 %)		
**Conduction disturbances**				
AV block II degree	1 (0,7 %)	0	0	
LBBB	1 (0,7 %)	0	0	
**Device dislocation**	5 (3,0 %)	1 (3,2 %)	1 (16,7 %)	Р1-2 = 0,87 р2-3 = 0,23 Р1-3 = 0,31
Hemolysis	1 (0,7 %)	0	0	Р1-2 = 0,67 Р1-3 = 0,94
Residual shunt more than 2 mm	3 (1,8 %)	0	1 (1,67 %)	Р1-2 = 0,13 р2-3 = 0,34 Р1-3 = 0,55
Procedure duration, min	73,0 ± 2,3	70,6 ± 4,3	111,7 ± 10,3	Р1-2 = 0,46 р2-3 = 0,12 Р1-3 = 0,05
X-ray time, min	20,8 ± 1,1	18,6 ± 3,3	36,8 ± 7,3	Р1-2 = 0,58 р2-3 = 0,05 Р1-3 = 0,02

Among patients with pmVSD inlet extension, four (2,4 %) experienced unsuccessful procedures. In the pmVSD outlet extension group, in two patients (6,5 %) procedure was unsuccessful, while all procedures in infracristal VSD group were successful.

## 7 Detailes of unsuccessful cases included

• One patient with pmVSD inlet extension who sustained TV chordae rupture during creation of arterio-venous loop;
• One patient experienced device-related severe AI due to aortic valve “pinching” by AVSO occluder disk;
• One case of device migration to the pulmonary artery occurring the day after the procedure. The patient had percutaneous device retrieval, but later refused any type of intervention).
• Three patients experienced device dislocation due to the underestimation of defect size. All of them had percutaneous device retrieval and underwent successful surgical repair.
Two patients with significant valve damage (tricuspid and aortic) underwent successful surgical repair. Both of them were categorised as a major complication as well. In patient with device related severe AI, intraoperative findings showed no structural damage of the aortic valve following device removal.

Overall, **major complications** occurred in 1 patient (0,6 %) in the pmVSD inlet extension group and 1 patient (3,22 %) in the pmVSD outlet extension group. No major complications were observed in the icVSD group. Patients with TR chordae rupture in pmVSD inlet extension group and severe device related severe AI were categorised as an unsuccessful procedure and described above.

In long term follow-up two significant adverse events were recorded (both were categorised as a major complications).

• One patient developed complete atrioventricular (AV) block and dilated cardiomyopathy two years after closure of pmVSD inlet extension with an 8 mm MVSO Amplatzer device (Abbott, USA). The patient's history included pulmonary artery (PA) banding with an adjustable band for multiple VSDs in infancy, followed by band balloon dilatation and transcatheter VSD closure at age two. The procedure was suboptimal because a residual gradient of 40 mmHg remained at the band site. Two years later the patient underwent device removal, surgical VSD repair, and permanent pacemaker implantation abroad for AV block. No later medical follow-up data are available.
• A second patient developed moderate aortic insufficiency (AI) immediately following transcatheter closure of an pmVSD. Over a four-year follow-up period the AI progressed to severe. The patient was listed for device explantation and a Ross procedure at a center abroad. Detailed review of imaging and intraoperative findings revealed no evidence of aortic valve “pinching.” The right coronary cusp appears to have been damaged during the intervention, suggesting the lesion was likely procedural rather than directly caused by the occluding device.

**Minor complications** occurred in 28 patients (16,87 %) with pmVSD inlet extension, 5 patients (16,12 %) with pmVSD outlet extension, and 3 patients (50 %) with infracristal VSD. The icVSD group demonstrated the statistically significant increase in minor complications (p_1-3_ = 0,03; p_2-3_ = 0,015). All minor complications are listed in Table 2 above.

## 8 Tricuspid regurgitation

New onset of mild TR was observed immediately after the procedure in 10 pts (6 %) with pmVSD inlet extension and 2 pts (6,5 %) with pmVSD outlet extension. Moderate TR was recorded in 14 patients (8,4 %) and 1 pt (3,2 %) in pmVSD inlet extension and pmVSD outlet extension groups correspondingly.

At the one-month follow-up, mild TR persisted in 12 patients (7,2 %) and 1 pt (3,2 %) in pmVSD inlet and pmVSD outlet extension group correspondingly, while moderate TR remained in only 3 patients (1,8 %) in pmVSD inlet extension group. No moderate TR was observed in outlet extension or infracristal VSD groups at this stage. No progression of TR was observed in later follow-up.

New onset of TR was recorded exclusively in patients who received when muscular VSD occluders (MVSO) or KONAR-MF devices. No correlation was identified between the incidence of TR and VSD anatomical subtypes, age of the patient, and procedural approach (antegrade or retrograde).

A weak negative correlation was noted between the use of Nit-occlud le VSD device and postprocedural TR (R = - 0,16, p = 0,027).

## 9 Aortic insufficiency

Aortic insufficiency of varying degree was observed in 7 patients (3,4 %). Five cases were classified as a mild, while one case of moderate AI progressed to severe over four-year follow-up period; aortic valve “pinching” was not documented. One patient developed severe AI shortly after the procedure and underwent surgical device explantation and VSD repair; aortic valve function was normal after device removal and did not require valve plasty. The procedure was categorised as unsuccessful and has been described previously. Aortic valve “pinching” by the left occluder disk was identified in 39 patients. The occurrence of de-novo AI demonstrated significant association with pre-existing AI (R = 0,43, p = 0,00004), pmVSD outlet extension (R = 0,22, p = 0,0016), and aortic valve “pinching” (R = 0,38, p = 0,0001). No correlation was found between de-novo AI after the procedure and patient age, size of the defect, type of the device, or approach to the closure.

With the introduction of the KONAR-MF device into clinical practice, we extended indications for percutaneous ventricular septal defect (VSD) closure to defects with a deficient aortic rim and aortic valve prolapse. The device's soft, thin, low-profile nitinol mesh adapts to the dynamic motion of the aortic valve leaflets and minimizes interference with leaflet mobility, permitting safer closure in anatomies previously considered high risk.

**Fig. 4:**
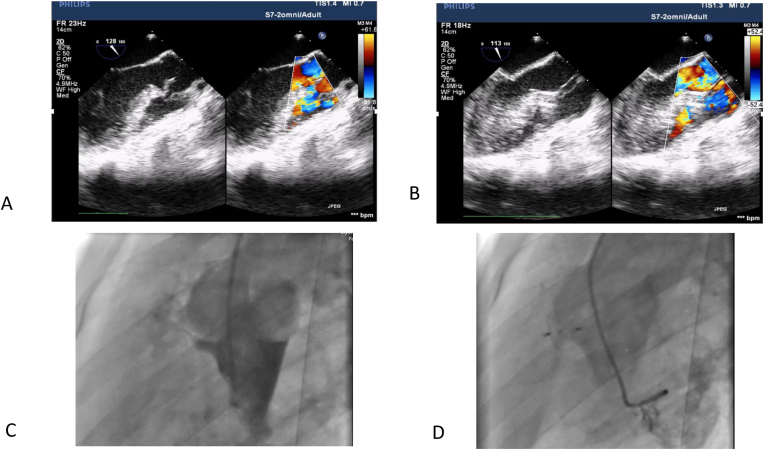
Subaortic VSD with right aortic cusp prolapse percutaneous closure.

Fig. 4 illustrates a representative successful percutaneous closure of a VSD complicated by aortic valve prolapse (Laubry-Pezzi syndrome). A 26-year-old woman was referred for treatment of a 4 mm perimembranous VSD with outlet (subaortic) extension; the right coronary aortic cusp prolapsed into the defect, producing moderate aortic insufficiency and left ventricular dilatation. She underwent percutaneous VSD closure with an 8–6 KONAR-MF device. At one-year clinical and echocardiographic follow-up, aortic regurgitation had regressed from moderate to mild and there was no residual intracardiac shunt.

**Fig. 5:**
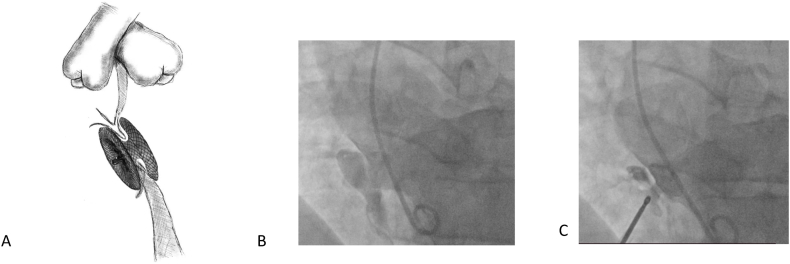
Device placed at the entry site of the aneurysm. **A**. Schematic diagram **B.** LV angiography show ventricular septal aneurysm with two exits **C.** VSD closed with 12 mm MemoPart MVSO. Patient experienced 2-d degree A-V block after procedure and required temporary pacemacker for 5 days.

## 10 Conduction disturbances

Three patients (1,48 %) experienced conduction disturbances. Two of them (one patient with left bundle branch block (LBBB) and one with 2nd degree of AV block) developed immediately after procedure were managed conservatively. The patient with 2-d degree A-V block received a temporary pacemaker and 0,5 mg/kg dexamethasone for 7 days. His angiography data are shown at Fig. 5. In these patients, conduction abnormalities were transient and no adverse events were recorded during later follow-up.

One patient developed complete AV block 2 years after 8 mm Amplatzer MVSO, requiring device removal and a permanent pacemaker. This patient was classified as having a major complication. Notably, no conduction abnormalities were observed immediately after the procedure in this case. In all patients who experienced conduction disturbances, the device either stent the defect directly (Fig. 3a) or was positioned at the entry site of the aneurysm (Fig. 5A–C). No genetic syndromes were observed in patients experiencing conduction abnormalities.

## 11 Residual shunt more than 2 mm

Significant residual shunt was observed in 4 pts (1,97 %). One patient required implantation of a second device due to a large residual shunt six months after the initial procedure. In 2 patients, residual shunt was reduced to minimal during later follow-up; one patient was lost from follow-up. No patients with moderate residual shunt experienced haemolysis.

## 12 Haemolysis

Haemolysis was observed only in one patient with pmVSD inlet extension closed with Nit-occlude Le VSD device. It resolved spontaneously within two days after the small in-device shunt disappeared.

## 13 Device dislocation

Device dislocation was observed in 7 patients (3,45 %). In one patient, the Nit-occlud le VSD coil migrated to the pulmonary artery the day after the procedure; it was retrieved via the femoral vein, but the patient declined further interventions or surgery. In three pts, the MVSO was exchanged for a larger one (2- for the larger MVSO and one for PDA occluder) during the same procedure. In another three patients, the actual defect exceeded 16 mm. All of them underwent percutaneous device retrieval and was referred for surgical repair, but one patient refused surgery. Notably, all these patients had defects deemed suitable for device (MVSO) closure based on TTE findings, with measured sizes ranging from 8 to 12 mm.

## 14 Small residual shunts

Small residual shunts remain a concern for infective endocarditis prophylaxis according to the ESC guidelines [20], which recommended antibiotic prophylaxis as long as even a small residual shunt persist near the synthetic material. The timing of residual shunt closure is presented in Table 3.

**Table 3:** Timing of residual shunt closure.

Discharge	3 months	6 months	12 months	36 months
149/203 (73,40 %)	73/197 (37,06 %)	55/192 (28,64 %)	38/178 (21,35 %)	22/137 (16,05) %)

Full neo-endothelization is generally expected to be completed within 6 months of device implantation. However, in our study residual shunt closure was observed as late as 36 months post-procedure. In our series, persistent residual shunts were documented at 36 months in twenty-two patients, indicating that closure may occur late in follow-up. Ten of them had Nit-occlud le VSD device, 10 patients had KONAR-MF device (8 without Dacron patch and 2 with Dacron patch), 2 patients had Amplatzer MVSO.

Residual shunt 6 months after procedure was more frequently seen in patients who underwent VSD closure with Nit-occlud le VSD device (R = 0,21, p = 0,0029); and less commonly in cases where the device stented the defect directly (Fig. 3a), exit site in aneurysm (Fig. 3c) or the entry site to the aneurysm (Fig. 3b) (R = −0,27, p = 0,0002).

All residual shunts observed in our study were small or in-device and did not require any interventions. No cases of infective endocarditis were recorded.

## 15 Radiation exposure and procedure duration

The overall radiation exposure was 23,49 ± 20,88 min and the average procedure duration were 81,66 ± 54,30 min. Both radiation exposure and procedure duration were significantly higher in the icVSD group. All relevant data are presented in Table 2.

## 16 Follow-up

Mean follow-up was 59,50 ± 35,60 (2–146) months. Seventeen patients were lost to follow-up (all of them are from pmVSD inlet extension group). Only two major adverse events occurred during long-term follow-up: one patient developed complete A-V block two years after the procedure, another patient with moderate AI post-procedure progressed to severe over four years. Both patients were followed outside of the country.

## 17 Discussion

The present study reports the evaluation and comparison of percutaneous closure outcomes across various anatomical types of perimembranous VSD using different devices (MVSO, PDO, coils and MF-KONAR). It also analyses complication and explores correlation between those complications and anatomical or procedural variables.

Based on our observations, no significant or only weak correlations were found between procedure success and VSD anatomy or defect size. Similarly, weak associations were noted between procedure success and the patients’ age, body surfase area (BSA), approach (antegrade vs retrograde) and type of device.

Some earlier reports have shown successful closure of subarterial ventricular septal defects (including pmVSD outlet extension and icVSD) using soft nitinol wire-mesh devices like ADO II, MFO KONAR or Nit-occlud le VSD, which accommodate aortic valve movement. However, long-term complications involving aortic valve damage remain unexplored. [[5], [6], [7]]. In our views severe aortic valve “pinching” accompanied by more than trivial AI should warrants device removal. According to the reports by *Quiman Li* and *Raimond N Haddad,* predictors of AI progression after device closure include: distance from aortic valve less than 2 mm, retrograde approach, pre-existing aortic valve pathology (e.g., bicuspid valve, AI, or aortic valve prolapse), prolonged fluoroscopic time and use of larger devices [21,22]. De-novo AI in our study was associated with pre-existing AI (R coefficient = 0,43, p = 0,00004), pmVSD outlet extension (R coefficient = 0,22, p = 0,0016), and aortic valve “pinching” (R coefficient = 0,38, p = 0,0001). Considering these correlations and those identified in previous reports, we suggest that severe aortic valve “pinching” should be avoided. In cases where aortic “pinching” is present, it may be necessary to change the approach from retrograde to antegrade to allow proper assessment of aortic valve function. Additionally, the use of AVSO and ADO I devices should be avoided for subarterial VSDs (pmVSD with outlet extension and icVSDs). *Mohammadreza Edraki* et al. reported that the incidence of AI in late follow-up after percutaneous closure in patients with deficient aortic rim did not differ in the surgical group [23].

New onset of TR is commonly seen after pmVSD closure due to the proximity to the tricuspid valve, but it often diminished or resolves over time [5,11,[24], [25], [26]]. In our study, 12 pts (5,91 %) experienced mild TR and 15 pts (7,39 %) – moderate TR. At the one-month follow-up, this had reduced to 3 pts (1,48 %) with moderate TR and 13 pts (6,4 %) with mild TR. Moreover, none of the patients required any interventions in follow-up. We attribute the decrease of the TR degree to the reduction of chordae tension over time or misinterpretation of left ventricle-to-right atrium in-device shunt. *M. Szkutnik and S.A. Qureshi et* all suggested that a higher incidence of new onset of TR is related to the longer waist of MVSO devices interfering with tricuspid valve motion [27,28]. *Diadong Jiang at all.*in their latest work concludes that lower body weight, larger defect outlet diameter, bigger right disc size, higher right disc diameter/body weight ratio, and longer procedure time (all *P* < 0.05) were associated with postprocedural TR [26]. Therefore, devices with large right disc should be avoided in litter patients. According to the previous statement an ideal device for pmVSDs with inlet extension should be ADO II, Cocoon aneurysmal type VSDO or ADO I device. In our experience, new onset of TR was observed only in patients with MVSO and KONAR-MF devices, despite the absence of a significant correlation between new onset TR and patient age, device type, defect location, or procedural approach. The most crucial factor, in our opinion, to avoid TR is the use of TEE guidance and careful search for the optimal device position. Often, several repositioning attempts are necessary.

Historically, patients undergoing transcatheter closure of pmVSD inlet or outlet extension and icVSD carries a high risk of complete AV block, with reported rates ranging from 2 to 22 % [12,14,[29], [30], [31]]. However, more recent studies have shown a decline in this risk to around 1 % [5,13,24]. Reported predictors of complete AV block includes: device oversizing >2 mm beyond defect diameter, young patient age, genetic syndromes and thin waist occluders [13]. Chang Bian et all. Reported four cases of conduction disturbances following pmVSD with aneurysm device closure; in all cases devices were placed at the entry site of the aneurysm [31]. Their findings suggest this location increases the risk of conduction abnormalities. Considering this, we have avoided thin-waist and asymmetric devices, preferring softer options. Two patients in our study experienced transient conduction disturbances. Both were treated with corticosteroids for 1–2 weeks; one required temporary pacing. (this device was placed at the aneurysm's entry site). One patient developed complete A-V block two years post-procedure. The child had a Swiss-cheese type VSD with an adjustable band (later partially dilated with balloon). Contributing factors likely included young age (3-year-old), residual high right ventricle pressure stressing the conduction tissue, and a high device size-to-weight ratio.

## 18 Study limitation

The main limitation of the study is retrospective (complete availability of the data we can access from the records is not in our control). In some patient's long-term follow-up data was collected by the interview and assessment of the medical examination made in others institution (sometimes not even in our country).

## 19 Conclusions

Our study demonstrates that the indications for pmVSD closure can be safely expanded to pmVSD with outlet extension with deficient aortic rim and icVSD even in the presence of aortic valve prolapse and up to moderate AI without serious long-term complications. Procedural success showed no significant association with any evaluated variables. The complication rate did not significantly differ in more complex defects, such as pmVSD with outlet and icVSD. Ifracristal VSD was associated with longer fluoroscopy time and procedure duration. Aortic rim less than 2 mm and the presence of aortic valve prolapse are no longer absolute contraindications for percutaneous VSD closure in selected patients, even when using symmetric devices.

## CRediT authorship contribution statement

**Nataliia Yashchuk:** Writing – original draft, Visualization, Resources, Formal analysis, Data curation, Conceptualization. **Igor Ditkivskyy:** Supervision, Resources, Methodology, Formal analysis, Data curation, Conceptualization. **Denys Voloshyn:** Visualization, Data curation. **Bogdan Cherpak:** Supervision, Methodology, Investigation, Formal analysis, Data curation, Conceptualization. **Yuliia Yermolovych:** Visualization, Formal analysis, Data curation.

## Declaration of competing interest

The authors declare that they have no known competing financial interests or personal relationships that could have appeared to influence the work reported in this paper.
